# Function of brain-derived neurotrophic factor in the hypothalamus: Implications for depression pathology

**DOI:** 10.3389/fnmol.2022.1028223

**Published:** 2022-11-16

**Authors:** Anita E. Autry

**Affiliations:** ^1^Dominick P. Purpura Department of Neuroscience, Albert Einstein College of Medicine, Bronx, NY, United States; ^2^Department of Psychiatry and Behavioral Sciences, Albert Einstein College of Medicine, Bronx, NY, United States

**Keywords:** BDNF, TrkB, depression, hypothalamus, corticotropin releasing hormone, vasopressin, oxytocin, stress

## Abstract

Depression is a prevalent mental health disorder and is the number one cause of disability worldwide. Risk factors for depression include genetic predisposition and stressful life events, and depression is twice as prevalent in women compared to men. Both clinical and preclinical research have implicated a critical role for brain-derived neurotrophic factor (BDNF) signaling in depression pathology as well as therapeutics. A preponderance of this research has focused on the role of BDNF and its primary receptor tropomyosin-related kinase B (TrkB) in the cortex and hippocampus. However, much of the symptomatology for depression is consistent with disruptions in functions of the hypothalamus including changes in weight, activity levels, responses to stress, and sociability. Here, we review evidence for the role of BDNF and TrkB signaling in the regions of the hypothalamus and their role in these autonomic and behavioral functions associated with depression. In addition, we identify areas for further research. Understanding the role of BDNF signaling in the hypothalamus will lead to valuable insights for sex- and stress-dependent neurobiological underpinnings of depression pathology.

## Introduction

Neurotrophins and their receptors have long been implicated in the pathophysiology of psychiatric diseases. This family of secreted growth factors includes brain-derived neurotrophic factor (BDNF), neurotrophin-3 (NT-3) and neurotrophin-4/5 which all bind to receptor tyrosine kinases Trk-B (Ntrk2) and P75_NTR_. In both human and animal studies, altered levels of neurotrophins, particularly BDNF, and their receptors have been implicated in Major Depressive Disorder, Schizophrenia, drug addiction, Post-traumatic Stress Disorder, and other disorders (Autry and Monteggia, 2012). Major Depressive Disorder (MDD) is currently the leading cause of disability worldwide, so understanding the biological and neuronal mechanisms of this serious mood disorder is a critical and urgent need (Friedrich, 2017). According to the DSM-5, MDD is characterized by symptoms of daily depressed mood, diminished pleasure, psychomotor slowing, changes in weight or sleep patterns, decreased energy or fatigue, reduced concentration or decisiveness, and recurrent thoughts of death (American Psychiatric Association[APA], 2022). A preponderance of clinical and experimental data indicates an essential role for BDNF in the pathophysiology of depression, leading to the “Neurotrophin Hypothesis of Depression” (Chen et al., 2001; Dwivedi et al., 2003; Duman and Monteggia, 2006; Sen et al., 2008; Dunham et al., 2009; Dwivedi, 2009; Tripp et al., 2012). Research on this hypothesis has primarily been focused on BDNF signaling in the cortex, hippocampus, and striatum as previously reviewed (Duman and Monteggia, 2006; Martinowich et al., 2007; Autry and Monteggia, 2012; Castren and Kojima, 2017; Yang et al., 2020). However, the hypothalamus is responsible for many of the functions that are altered in the pathophysiology of depression including homeostatic regulation of bodily functions including sleep, thermoregulation, hunger, thirst, energy balance, osmoregulation, and cardiovascular functions. In addition, the hypothalamus plays a fundamental role in hypothalamic-pituitary adrenal axis (HPA) responses to acute or chronic stress, a critical risk factor for depression etiology. Finally, the hypothalamus in known to have a key role in social behaviors, a domain of depression pathology related to interest and pleasure. Here we review the role of BDNF in the hypothalamus and discuss important future research avenues to understand the implications of BDNF signaling in the hypothalamus for depression.

## Brain-derived neurotrophic factor and receptor expression in the hypothalamus

Brain-derived neurotrophic factor is highly expressed across regions of the hypothalamus (Castren et al., 1995; Marmigere et al., 1998; Aliaga et al., 2002; Rage et al., 2002). Allen Brain Atlas (brain-map.org) data shows BDNF expression in a P56 male mouse in the medial preoptic area (MPOA), the paraventricular hypothalamus (PVH), the arcuate nucleus (Arc), the ventromedial hypothalamus (VMH), the lateral hypothalamus (LH), and the ventral premamillary nucleus (PMV). The Human Protein Atlas (proteinatlas.org) demonstrates expression in adult men and women in the Arc, the dorsomedial nucleus (DMH), the LH (male subjects only), the mammillary bodies, the PVH, the MPOA, and the supraoptic nucleus (SON) (Sjostedt et al., 2020). Tropomyosin-related kinase B (TrkB) (Ntrk2) is detected across all brain regions in human, pig, and mouse tissue samples (Sjostedt et al., 2020).

## Transcriptional regulation of brain-derived neurotrophic factor in the hypothalamus

A single BDNF protein is generated by the coding exon of BDNF, but it can be transcribed from nine promoters resulting in around 22 distinct transcripts (Aid et al., 2007; Pruunsild et al., 2007; West et al., 2014). These promoters are differentially active across brain regions and some show strong activity-dependent regulation (Timmusk et al., 1993). The hypothalamus largely expresses transcripts generated by BDNF promoters I and II (Maynard et al., 2016; You et al., 2020).

## Secretion of brain-derived neurotrophic factor

Brain-derived neurotrophic factor has a variety of neuronal functions across development and in the adult. In the developing brain, BDNF is responsible for neurite outgrowth and cell survival (Pillai, 2008; Yoshii and Constantine-Paton, 2010). In the mature brain, BDNF promotes both excitatory and inhibitory neurotransmission, pre- and post-synaptically, modulating spontaneous and evoked signaling (Schuman, 1999; Tyler et al., 2006; Waterhouse and Xu, 2009; Park and Poo, 2013; Edelmann et al., 2014). Recently, it has been demonstrated that BDNF is predominantly stored in dense-core vesicles in presynaptic terminals, but physiologically relevant secretion of BDNF has also been shown to occur at postsynaptic sites as well as from astrocytes and microglia (Chen et al., 2005; Dean et al., 2009; Lessmann and Brigadski, 2009; Parpura and Zorec, 2010; Dieni et al., 2012; Parkhurst et al., 2013; Harward et al., 2016; Song et al., 2017).

Brain-derived neurotrophic factor can be released in a non-regulated manner *via* the constitutive secretory pathway (Brigadski et al., 2005), or secreted in an activity-dependent manner (Waterhouse and Xu, 2009; Brigadski and Lessmann, 2020). The most prevalent single nucleotide polymorphism of BDNF in humans is the Val to Met (val66met) polymorphism which prevents activity-dependent secretion (Egan et al., 2003). This SNP has been associated with increased risk or susceptibility for MDD (Gatt et al., 2009; Dwivedi, 2010; Frielingsdorf et al., 2010) and impaired cognition (Hariri et al., 2003) as well as impaired fear extinction evident in Posttraumatic stress disorder (PTSD) (Frielingsdorf et al., 2010; Felmingham et al., 2018; Rippey et al., 2022) in humans.

## Steroid regulation of brain-derived neurotrophic factor expression: Stress and sex differences

Steroid hormones, including estrogen, mineralocorticoids, and glucocorticoids, are known to regulate the expression of BDNF. The BDNF promoter is thought to contain both an estrogen response element (ERE) (Sohrabji et al., 1995) and a glucocorticoid response element (GRE) (Suri and Vaidya, 2013), though direct evidence is lacking and regulation may occur at the transcriptional level or at the level of translation, post-translational processing, or intracellular trafficking and secretion. Thus, BDNF expression is highly dependent on sex and physiological status, as will be discussed in detail below. Extant research has focused on how BDNF is regulated in the hippocampus by estrogens (Solum and Handa, 2002; Harte-Hargrove et al., 2013). However, there is little research characterizing how BDNF expression is affected by estrogen in the hypothalamus.

### Estrogen

Estrogen signaling in the brain can occur through uptake of peripheral estrogen (Baulieu and Robel, 1998), local synthesis of estrogen from cholesterol (Hojo et al., 2008), or conversion of androgen to estrogen *via* aromatase activity (Roselli, 2007). Aromatase was originally discovered in the hypothalamus where it is highly expressed in humans and rodents (Naftolin et al., 1971, 1972), illustrating that local synthesis of estrogen occurs in the hypothalamus *via* aromatization. Estrogen impacts many functions of the hypothalamus including reproductive behavior, temperature and energy balance, stress responses, and motivated behavior (Kelly et al., 2005). Research on the impact of estrogen’s effect on BDNF expression in the cortex and hippocampus reveal conflicting results, reporting a positive (Sohrabji et al., 1995; Gibbs, 1999; Jezierski and Sohrabji, 2001), negative (Singh et al., 1995; Gibbs, 1998, 1999; Cavus and Duman, 2003), or neutral impact (Jezierski and Sohrabji, 2000) of estrogen on the expression of BDNF. Studies in hypothalamic slice cultures reveal that acute or chronic treatment with estrogen does not alter BDNF mRNA levels (Viant et al., 2000). However, slice culture experiments may remove important circuit inputs for BDNF into hypothalamic structures, therefore further study is required. In addition, it is known that BDNF levels in the cortex and hippocampus fluctuate according to estrous cycle (Gibbs, 1998; Cavus and Duman, 2003). It is therefore likely that estrogen regulation of BDNF signaling is an important factor in hypothalamic functions, so understanding how estrogen and BDNF signaling interact in the hypothalamus across sex and physiological status is an area deserving of experimental attention.

### Glucocorticoids and stress

Glucocorticoids are well-known to impact BDNF expression in the hippocampus and cortex (Barbany and Persson, 1992, 1993; Schaaf et al., 1997, 1998; Hansson et al., 2000), however less is known about how glucocorticoids impact BDNF expression in the hypothalamus. Knockdown of glucocorticoid receptor (GR) in the PVH leads to increased protein levels of both BDNF and TrkB (Jeanneteau et al., 2012). In addition, acute or repeated restraint stress rapidly increases BDNF protein levels in the hypothalamus as well as BDNF transcription, particularly in the PVH and SON (Smith et al., 1995a; Rage et al., 2002). Together, these data suggest that hypothalamic BDNF expression is maintained by physiological glucocorticoid receptor activity, and changes in activity of the receptor through reduced or increased activation of the receptor will increase BDNF levels. TrkB activation by BDNF in the PVH leads to reduced surface expression of GABAa receptors (Hewitt and Bains, 2006), which likely contributes to increased excitability of PVH cells. Further characterization of how stress and stress hormones including glucocorticoids and mineralocorticoids modulate BDNF signaling in the hypothalamus in a sex-dependent manner is necessary.

Importantly, the impact of stress on BDNF expression in the hypothalamus is opposite to that observed in the hippocampus, where stress decreases BDNF expression (Smith et al., 1995b). This finding raises the possibility that stress and steroid hormones including glucocorticoids, estrogen, or others like progesterone (Zhang et al., 2007; Singh and Su, 2013) may interact in a brain-region or circuit-specific manner in adults or across developmental timespoints (Notaras and Van Den Buuse, 2020). For example, adolescent corticosterone treatment leads to cognitive impairment in female mice homozygous for the val66met mutation (Raju et al., 2022) and altered interneuron signaling in both sexes (Hill et al., 2020), and the val66met mutation leads to altered physiological responses in the hippocampus after acute stress in male mice (Musazzi et al., 2022). Furthermore, the val66met mutation reveals increased anxiety- and depression-related behavior after treatment with corticosterone (Caradonna et al., 2022). Therefore, study of the intersectional effects of glucocorticoids, stress, BDNF and BDNF mutations, and estrogen in specific nuclei of the hypothalamus are critical.

## Role of hypothalamic brain-derived neurotrophic factor signaling in homeostatic and social behaviors

### Cardiovascular responses and blood pressure

Depression is highly comorbid with cardiovascular disease, which is the number one cause of death in the United States (Hare et al., 2014; Tsao et al., 2022), likely due in part to the importance of stress as a risk factor in each disease. The PVH has a key role for integrating autonomic, neuroendocrine, and cardiovascular responses under physiological or stressful conditions (Dampney et al., 2005; Wamsteeker and Bains, 2010; Busnardo et al., 2014). Infusions of BDNF into the PVH increase mean arterial pressure (MAP) and heart rate (HR), mediated through TrkB receptors and angiotensin II receptor signaling (Schaich et al., 2016). Viral overexpression of BDNF in the PVH increases MAP and HR and alters stress recovery of HR and MAP (Erdos et al., 2015). These studies suggest that these effects are mediated through projections from the PVH to the nucleus solitary tract (NTS). TrkB signaling in the NTS has been associated with the anorexigenic effects of BDNF signaling, but there may be a critical role for BDNF signaling in this PVH to NTS projection for regulation of cardiovascular function as well (Spaeth et al., 2012). Thus, PVH BDNF signaling to NTS TrkB expressing cells may underlie stress-responsive cardiovascular responses which may become altered in depression due to adaptations after chronic stress. Further dissection of the role of BDNF and TrkB signaling in the hypothalamus and connected regions and how it may be affected by stress and sex will greatly enhance our understanding of cardiovascular responses in depression pathology.

### Body weight, feeding, and energy balance

Weight and appetite changes as well as fatigue are symptoms of depression. Several hypothalamic nuclei play a critical role in feeding, body weight, and energy balance (Lawrence et al., 1999). Human studies have revealed association of the val66met BDNF polymorphism with obesity, suggesting a clinical role for BDNF signaling in feeding and body weight regulation (Marques-Iturria et al., 2014; Martinez-Ezquerro et al., 2017; Goldfield et al., 2021), and this finding has recently been recapitulated in a mouse model (Ieraci et al., 2020). Recent studies in zebrafish also highlight the impact of feeding state and food availability on the regulation of BDNF, suggesting a conservation of BDNF’s function in food intake (Blanco et al., 2020). Previous comprehensive reviews have suggested a framework in which hypothalamic BDNF-TrkB signaling promotes satiety and maintains body weight and energy balance in the PVH *via* melanocortin 4 receptor (MC4R) signaling, in the VMH *via* leptin and glucose signaling, and in the DMH *via* unclear mechanisms (Noble et al., 2011; Rios, 2013). Here we will examine recent studies that provide updates to this proposed model.

#### Paraventricular hypothalamus

As expected, viral overexpression of BDNF in the PVH decreases body weight in male rats (Erdos et al., 2015). Activation of BDNF-expressing neurons of the PVH using a chemogenetic approach leads to suppressed food intake in male and female mice in both a fed and fasted state (Wu and Xu, 2022). The same manipulation also raises respiratory exchange ratio (RER), and oxygen consumption in both sexes (Wu and Xu, 2022).

Knockdown of BDNF in the PVH using the Sim1 promoter results in higher body weight, size, fat pad mass, increased food intake during adulthood, and reduced energy expenditure in both male and female mice (An et al., 2015). Sim1 is expressed during late embryogenesis and early postnatally in the PVH, SON, and posterior hypothalamus with very little extra-hypothalamic expression (Balthasar et al., 2005). These changes are recapitulated by knocking out BDNF in the PVH in adults using a viral approach (An et al., 2015).

Similarly, deletion of TrkB in the PVH using Sim1-Cre leads to obesity (An et al., 2020). PVH neurons that express TrkB do not overlap with the population that expresses BDNF or other known appetite-suppressing neuron types in the PVH, although chemogenetic manipulations reveal that they are involved in anorexigenic behavior (An et al., 2020). Deletion of PVH TrkB from projections to either the lateral parabrachial nucleus or to the ventromedial hypothalamus lead to hyperphagia and obesity (An et al., 2020). Interestingly, the authors rule out the PVH TrkB projection to NTS as being critical for feeding, but other projections from PVH to NTS may be essential (An et al., 2020).

#### Dorsomedial, ventromedial, and lateral hypothalamus

Chemogenetic manipulations of TrkB-expressing neurons of the DMH reveals that activation of these cells reduces intake during the active period while inhibition uncovers increased feeding during the inactive period (Liao et al., 2019). Deletion of TrkB from the DMH leads to increased body weight and food intake, increased blood glucose levels, and reduced energy expenditure and activity levels (Liao et al., 2019). Selective deletion of BDNF in the adult ventromedial hypothalamus also leads to increased body weight and hyperphagia (Unger et al., 2007; Yang et al., 2016). This phenotype was recapitulated by knocking down hypothalamic BDNF using the Nkx2.1 promoter (Yang et al., 2016). Knockdown of BDNF expression *via* exon I promoter activity, but not exon IV or VI, leads to increased body weight, body length, and mass of adipose tissue (You et al., 2020). This manipulation leads to larger reductions in BDNF protein in the lateral hypothalamus relative to nearby dorso- and ventromedial hypothalamic nuclei, and indeed the phenotype can be rescued by infusion of a TrkB agonist into the lateral hypothalamus (You et al., 2020). It was previously shown that global knockdown of TrkB by around 25% leads to hyperphagia and obesity soon after weaning (Xu et al., 2003). Similarly, knockdown of TrkB in astrocytes in the ventromedial hypothalamus using the glial fibrillary acidic protein (GFAP) promoter recapitulates many effects of BDNF deletion in the VMH including increased body weight, increased food intake, and hyperphagia in male and female mutants (Ameroso et al., 2022).

Recent experiments have also provided new insights into the function of BDNF in the arcuate nucleus. Anatomical studies reveal that TrkB is expressed in arcuate nucleus neurons, but do not overlap with proopiomelanocortin (POMC) or neuropeptide Y (NPY)-expressing cells (Liao et al., 2015). Food deprivation leads to a decrease in mRNA expression and number of cells immunoreactive for BDNF in the arcuate nucleus of male mice while short term access to a high fat diet increased the number of BDNF immunoreactive cells in the VMH (Gilland and Fox, 2017). However, mechanistic evidence is lacking in terms of the role of BDNF-TrkB signaling in the arcuate nucleus in control of food intake and body weight, so further research is needed.

Altogether, many of these studies suggest that BDNF-TrkB signaling among hypothalamic nuclei involved in feeding regulate food intake and body weight. However, while we can infer some microcircuitry, the role of secreted BDNF and TrkB signaling cannot be determined without manipulations that impact synaptic BDNF levels.

### Psychomotor activation

Depression symptoms include changes in physical activity levels. Many mouse strains with loss of BDNF show changes in locomotor activity as described in detail in the following section. The global heterozygous knockout (BDNF^±^) shows increased locomotion in mice with normal weights while mice that become heavier due to hyperphagia display normal levels of locomotion (Kernie et al., 2000). Amphetamine-potentiated psychomotor activation is stronger in BDNF^±^ mice compared to wild-type mice (Dluzen et al., 2001; Saylor and Mcginty, 2008). Under normal physiological conditions, male mice with conditional knockout of BDNF in forebrain regions using various promoters [GFAP, calcium-calmodulin-dependent kinase II (CamKII), and NSE] display increased locomotion in a novel environment, while females do not (Monteggia et al., 2004, 2007; Autry et al., 2009). In contrast, female BDNF knockouts using the neuron-specific enolase (NSE) promoter do show a reduction in psychomotor activity after stress (Autry et al., 2009). These data illustrate that while females may not show baseline changes with loss of BDNF, there could be important sex-differences underlying stress responses. Notably, deletion of BDNF in the hypothalamus using either the Sim1 promoter or viral knockdown in the PVH also reduces locomotion in males and females (An et al., 2015). Deletion of BDNF in the ventromedial hypothalamus has been observed to have either no impact on locomotion (Unger et al., 2007) or to lead to a reduction in locomotor activity (Yang et al., 2016). On the other hand, viral overexpression of BDNF in the PVH in male rats increases locomotor activity (Erdos et al., 2015). Chemogenetic activation of BDNF-expressing neurons in the PVH also leads to increased locomotion in both male and female mice (Wu and Xu, 2022). Altogether, these data show BDNF signaling in the hypothalamus plays a critical sex- and stress-dependent role in the coordination of satiety and body weight maintenance in part by increasing psychomotor activity.

### Thermogenesis

It has been proposed that whole-body cryotherapy or hyperthermia may be an important non-invasive intervention to relieve symptoms of depression (Rymaszewska et al., 2008; Janssen et al., 2016). Knockdown of BDNF expressed from the promoter for Exon I reduced core body temperature at both room temperature and after cold stress (You et al., 2020). This effect was rescued by infusion of a TrkB agonist into the lateral hypothalamus (You et al., 2020). Stimulation of PVH BDNF neurons leads to increased core body temperature and brown adipose tissue thermogenesis (Wu and Xu, 2022). Neurons responsible for warm sensing in the MPOA express BDNF and pituitary adenylate cyclase-activating polypeptide (PACAP) and translating ribosome affinity-purification from each population demonstrates that these peptides are co-expressed in a subset of MPOA neurons (Tan et al., 2016). Optogenetic stimulation of BDNF neurons in the MPOA decreases body temperature and reduces brown adipose tissue thermogenesis (Tan et al., 2016). Chemogenetic activation of TrkB-expressing neurons in the DMH increases body temperature and adipose tissue thermogenesis, with opposite effects induced by inhibition (Houtz et al., 2021). These effects appear to be mediated by TrkB-expressing DMH neurons that project to both the PVH and the preoptic area (Houtz et al., 2021). While it is likely that optogenetic stimulation of neurons expressing BDNF will lead to BDNF release, BDNF release with this manipulation has not been directly demonstrated. Assessment of the impact of optogenetic manipulation on synaptic BDNF release will be a critical control to interpret effects of optogenetic stimulation. Altogether, this evidence suggests that BDNF signaling in the hypothalamus is essential for thermogenesis as well as temperature preference behaviors.

### Social behaviors

Depression is associated with reduced interest in social interactions. Preclinical evidence has consistently associated BDNF signaling with alterations in social behavior. Both prosocial and aggression behavior are mediated through hypothalamic nuclei (Kruk, 1991; Lin et al., 2011; Kohl et al., 2017; Walsh et al., 2022). BDNF heterozygous males show increased aggression (Lyons et al., 1999), and this effect is recapitulated in a conditional forebrain-specific knockdown of BDNF using a CamKII promoter (Rios et al., 2001), or a KA promoter that knocks down BDNF in the hippocampus and cerebellum (Ito et al., 2011). This hyperaggressive phenotype is apparent with either fetal or postnatal loss of BDNF, although a more robust phenotype emerges with early deletion (Chan et al., 2006). Loss of BDNF expression from promoters I and II, which predominate in the hypothalamus, leads to enhanced aggression in male mice (Maynard et al., 2016). Conversely, selective loss of BDNF in the DMH or VMH does not impact aggression (Unger et al., 2007). However, it is critical to note that differences in aggression may be regulated cell-autonomously through TrkB signaling; for example, deletion of BDNF in the dorsal raphe nucleus does not affect aggression behavior while loss of TrkB in this nucleus increases aggression (Adachi et al., 2017). Thus, it will be important to determine the effect of receptor loss in the DMH or VMH to uncover the role of BDNF signaling in each nucleus of this aggression circuit. Altogether, these data suggest that further examination of the role of BDNF in specific hypothalamic circuits and cell populations can refine our understanding of how BDNF signaling modulates aggressive behavior.

There is also evidence that BDNF signaling may also have a role in female-typical social behavior. In a communal nesting experiment, pups that experienced the highest levels of maternal care showed a 2-fold increase in hypothalamic BDNF protein levels while mice experiencing the least maternal care had a trend toward reduced hypothalamic BDNF levels in adulthood relative to mice reared in standard conditions (Branchi et al., 2013). Further research shows that loss of BDNF expression *via* the exon I promoter leads to reduced infant-directed behaviors in virgin and lactating female mice (Maynard et al., 2018). The authors attributed this caregiving deficit to reduced expression of oxytocin in the PVH (Maynard et al., 2018). Selective deletion of the TrkB receptor in oxytocin-expressing PVH neurons recapitulates these deficits in infant-directed behavior (Maynard et al., 2018). These data strongly implicate BDNF signaling in maternal care, a process in which experience dependent plasticity is essential (Fuentes et al., 2022). Future studies focused on the role of BDNF and TrkB in other hypothalamic nuclei associated with parenting such as the anteroventral periventricular nucleus and the MPOA are needed (Kohl et al., 2017).

## Brain-derived neurotrophic factor signaling in specific hypothalamic cell populations

Given the diverse functions of BDNF signaling, it is critical to develop an understanding of how BDNF impacts specific cell types in the hypothalamus. Evidence reviewed thus far suggests a critical role for BDNF in the PVH for both homeostatic functions and social behaviors. Chronic intracerebroventricular infusion of BDNF increases expression of corticotropin releasing hormone and vasopressin in the PVH (Naert et al., 2006). TrkB is highly expressed in oxytocin neurons of the PVH, and loss of BDNF expression from exon I reduces expression of oxytocin in the PVH (Maynard et al., 2018). Overexpression of BDNF in Sim1-positive neurons leads to 1.5-fold increased expression of corticotropin releasing hormone (CRH) in the PVH (Jeanneteau et al., 2012). Furthermore, TrkB heterozygous knockdown leads to reduced (CRH) expression in the PVH (Jeanneteau et al., 2012). Altogether, these studies suggest a critical link between BDNF signaling and appropriate development and function of PVH CRH cells. However, infusion, overexpression, or deletion cannot give true insights into dynamics of endogenously released BDNF, so further studies that probe the impact of interfering with actions of released BDNF such at the BDNF val66met polymorphism or using TrkB-Fc would provide increased specificity for interpreting the impact of BDNF signaling on this critical circuit node.

The hypothalamus contains several neuropeptide-expressing or sensing cell populations that control feeding including agouti-related peptide-expressing neurons and pro-opiomelanocortin-expressing cells of the arcuate nucleus, as well as oxytocin- and melanocortin receptor 4 expressing cells of the PVH (Atasoy et al., 2012; Krashes et al., 2013; Shah et al., 2014; Mandelblat-Cerf et al., 2015). Here, we have reviewed a substantial body of literature illustrating the dramatic and bidirectional impact of hypothalamic BDNF signaling on feeding behavior and weight regulation. Together with these recent advances in a cell-type specific understanding of feeding control, it becomes apparent that a critical avenue for future research is focus on BDNF and TrkB signaling in these defined hypothalamic cell-types.

## Conclusion

**FIGURE 1:**
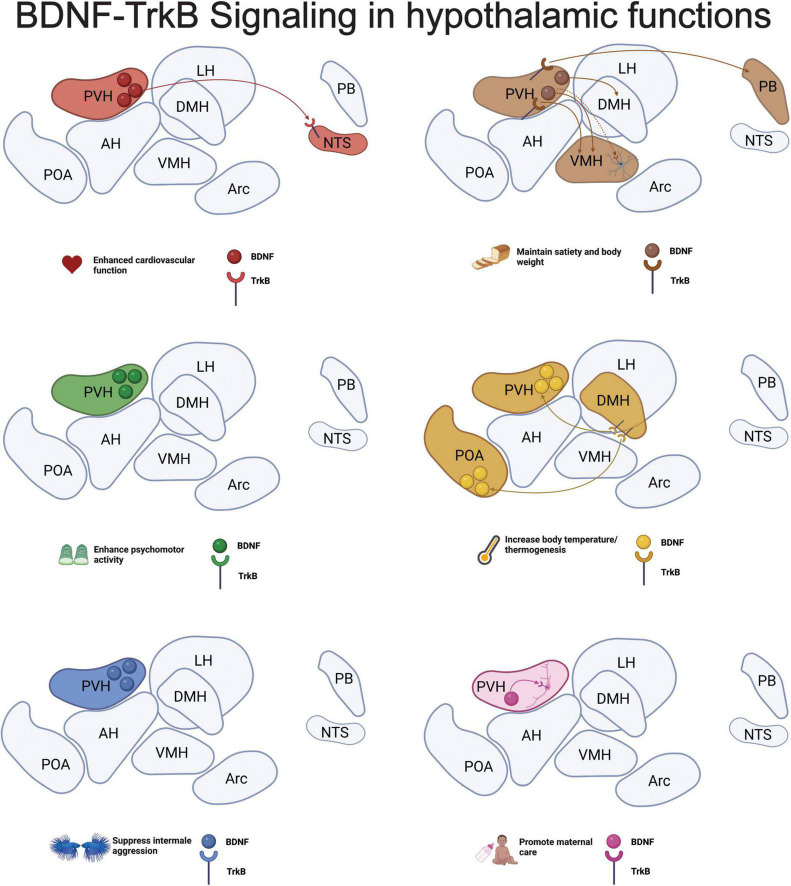
Brain-derived neurotrophic factor (BDNF)-tropomyosin-related kinase B (TrkB) signaling in hypothalamic functions. Here we summarize the current findings on the role of BDNF and TrkB signaling in hypothalamic nuclei discussed in each section of this review. Much of the research has been done using knockout and infusion studies, so cell-type information is largely missing. However, research shows that astrocytes expressing TrkB in the ventromedial hypothalamus (VMH) are critical for maintaining body weight and satiety and oxytocin-expressing neurons require TrkB signaling to promote infant care in mothers. Altogether, we see a critical role for BDNF-TrkB signaling both within the paraventricular hypothalamus (PVH) and in the afferent and efferent projections from the region in all hypothalamic functions. Representation of hypothalamic nuclei was adapted from (Hajdarovic et al., 2022) and the schematic was developed using Biorender.

Here we have reviewed research relevant for the role of hypothalamic BDNF signaling in depression-related symptomology. Overall, there are many promising results illustrating the crucial role of BDNF signaling in the hypothalamus is involved in cardiovascular responses, feeding and body weight, psychomotor activity, thermogenesis, and social behaviors (Figure 1). Currently, major gaps exist for the study of stress responses in a sex-dependent manner, which is critical given the regulation of hypothalamic BDNF signaling by glucocorticoids and estrogen. Importantly, depression rates are nearly double in women compared to men, so this area of inquiry is essential for basic neurobiology insights into this sex difference (Brody et al., 2018). In addition, further studies dissecting the circuit- and cell-type specific role of BDNF and TrkB signaling in the hypothalamus are needed. Examining these open avenues in the future will greatly enhance our understanding of depression pathology.

## Author contributions

The author confirms being the sole contributor of this work and has approved it for publication.
